# Book Reviews

**Published:** 1891-04

**Authors:** 


### Book Reviews.

A Guide to the Practical Examination of Urine. For the use of physicians and students. By James Tyson, M. D. Seventh edition.

That this work should have reached a seventh edition speaks for itself of the value of the book ; more in its praise could not be said.

It is published by P. Blakiston, Son & Co., Philadelphia.

---

Essentials of Minor Surgery and Bandaging, with an appendix on Venereal Diseases. By Edward Martin, A. M., M. D. This is Saunders' Question Compend No. 12.

This little book, like all others of this series, seems to fill a long felt want, and will doubtless be fully appreciated.

---

Saunders' Question Compend, No. 18, is the Essentials of Pharmacy, by Prof. L. E. Sayer, Ph. G.

This is the latest of this series, and will, we are sure, meet with a hearty reception.

The little books cannot be too highly recommended to the busy practitioner and to students.

